# mHealth Technology Experiences of Middle-Aged and Older Individuals With Visual Impairments: Cross-Sectional Interview Study

**DOI:** 10.2196/52410

**Published:** 2023-12-25

**Authors:** Soyoung Choi, Md Refat Uz Zaman Sajib, Jenna Manzano, Christian Joseph Chlebek

**Affiliations:** 1 Department of Kinesiology and Community Health University of Illinois at Urbana-Champaign Urbana, IL United States; 2 College of Liberal Arts and Sciences University of Illinois at Urbana-Champaign Urbana, IL United States

**Keywords:** aging, mobile health, older adults, technology, visual impairment, wearables, wearable, vision, visual, qualitative analysis, health behavior, mHealth, mHealth technology, digital technology, medical application, application, app, applications, usage, well-being, cross-section interview, interview, interviews, tracking, health data, symptom monitoring, monitor, monitoring, symptom, symptoms, physical activity, walking, routine, mobile phone

## Abstract

**Background:**

Current mobile health (mHealth) technology is predominantly designed with a visual orientation, often resulting in user interfaces that are inaccessible to visually impaired users. While mHealth technology offers potential for facilitating chronic illness management and enhancing health behaviors among visually impaired older populations, understanding its usage remains limited.

**Objective:**

This qualitative research aimed to explore the mHealth technology experiences of middle-aged and older individuals with visual impairments including the accessibility and usability issues they faced.

**Methods:**

The qualitative exploration was structured using the mHealth for Older Users framework. Cross-sectional interviews were conducted via Zoom between June 1 and July 31, 2023, using an interview protocol for data collection. A thematic analysis approach was employed to analyze the transcribed interview scripts.

**Results:**

Of the 7 participants who took part in the Zoom interviews, 3 were men and 4 were women, with ages ranging from 53 to 70 years. Most participants adopted mHealth apps and wearable devices for promoting health. They exhibited 3 distinct adoption patterns. Seven themes were emerged from the perceived challenges in using mHealth technologies: (1) a scarcity of accessible user manuals, (2) user interfaces that are not visually impaired-friendly, (3) health data visualizations that are not accessible, (4) unintuitive arrangement of app content, (5) health information that is challenging to comprehend, (6) cognitive overload caused by an excess of audible information, and (7) skepticism regarding the accuracy of health records. mHealth technologies seem to positively affect the health and health management of participants.

**Conclusions:**

Design considerations for mHealth technologies should consider individuals’ disabilities and chronic conditions and should emphasize the importance of providing accessible manuals and training opportunities when introducing new mHealth solutions.

## Introduction

Visual impairment is a permanent reduction in visual capabilities that affects task performance and cannot be corrected with glasses, contact lenses, or medical interventions [1]. Due to the normal aging process, as well as congenital disabilities, accidents, or ophthalmological conditions, individuals can experience a decline in near visual acuity and visual field [2]. Specifically, the term “Low vision” refers to a level of visual impairment that cannot be corrected by glasses, contact lenses, medication, or surgery, significantly affecting an individual’s ability to perform daily routines [3]. Meanwhile, “blindness” describes a more severe visual impairment, characterized by a complete inability to perceive light [3]. About 253 million people live with visual impairment worldwide, and in the United States, the prevalence of low vision and blindness is approximately 3% and 1%, respectively [4]. The substantial impact of visual impairment underscores the importance of public health approaches to meet the varying health care needs of those affected by low vision and blindness.

Mobile health, also known as mHealth, encompasses the usage of mobile devices, including mobile phones, patient monitoring equipment, and other wireless technologies, in medical and public health practices [5]. mHealth technologies enable individuals to monitor their chronic conditions in real time [6] and offer consistent medical support for both patients and their family caregivers [7]. However, the usage of mHealth technologies is inconsistent and not as prevalent among older individuals [8]. According to the previous studies, an older individual’s continued use of mHealth technologies is significantly influenced by their perceived satisfaction as well as its perceived ease of use and apparent advantages [9,10]. In addition, mismatch in health-monitoring needs compared to younger groups, designs not tailored for older adults, poor usability, lack of adequate motivation strategies, health-related challenges posed by chronic illnesses, varying attitudes toward the technology can affect the mHealth technology usage in older populations [11,12].

In the United States, smartphone ownership among adults aged 65 years and older is 53% and they generally show low acceptance of wearable devices [13]. Nevertheless, older individuals who do use smartphones are significantly more inclined to express interest in wearable health devices [14]. In general, older individuals use mHealth technologies to enhance health-related activities; and individuals with chronic health conditions use their smartphone apps and wearable devices for monitoring health goals, making informed health decisions, and participating in health discussions with their health care providers [15,16]. However, there is a gap in understanding regarding the ways in which visually impaired older individuals use mHealth apps and wearable devices to better their health and well-being. Since most mHealth technologies require visual abilities from the user, it is anticipated that older individuals with visual impairments will face greater constraints in using these tools for health management, and the number of such users is also expected to be very limited.

This study aimed to bridge the knowledge gap among diverse groups benefiting from mHealth technology. It specifically focused on exploring the experiences of middle-aged and older individuals with visual impairments, including those with low vision and blindness, in using mHealth technologies, particularly mHealth apps and wearable devices. For our research, we employed a literature-based framework known as mHealth for Older Users (MOLD-US) [12] to serve as a lens for comprehensively understanding the experiences of middle-aged and older adults with visual impairments in using mHealth apps and wearable devices. The selected framework has been developed to examine the age-related barriers that impact the usability of mHealth apps among older adults [12]. By grounding our research in this model, we developed the following research questions:

- RQ1. How do middle-aged and older individuals with low vision or blindness use mHealth technologies, including mHealth apps and wearable devices, for managing their health?
- RQ2. What are the obstacles that middle-aged and older individuals with low vision or blindness face when using mHealth apps and wearable devices?
- RQ3. How do mHealth apps and wearable devices impact the health and health management of middle-aged and older adults with low vision or blindness?

## Methods

### Theoretical Model

The theoretical underpinnings of this research are grounded in the “MOLD-US” framework [12]. The authors of the framework performed an exhaustive review of the literature to pinpoint studies that investigate the barriers older adults face in using mHealth apps. This framework is organized into 4 primary types of age-related barriers: physical (eg, motor skills, dexterity, and physical limitations), cognitive (eg, memory, attention, and problem-solving abilities), sensory (eg, vision, hearing, and touch), and psychological (eg, attitudes, beliefs, and social support). Each of these categories encompasses elements that could influence the ease of use of mHealth apps among older populations. Based upon this theoretical framework, the interview questions were developed. Furthermore, the research team conducted an analysis of the interview scripts to pinpoint challenges faced by visually impaired individuals while using mHealth apps or wearable devices.

### Research Design

A qualitative exploratory study was conducted using cross-sectional interviews via Zoom (Zoom Video Communications, Inc).

### Ethical Considerations

All research procedures and protocols were submitted to the institutional review board at the University of Illinois and received approval, confirming our adherence to ethical standards (23791). Due to participants’ inability to sign documents, informed consent was obtained verbally. Identifiable information was removed from the collected data to ensure confidentiality, and access to these digital files was restricted to members of the research team. To express our gratitude for their contributions, participants were given a US $40 Amazon e-gift card, which was promptly emailed to them following their interview.

### Participant Recruitment

In this study, we engaged 7 older adults with visual impairments who are experienced in using mHealth apps and wearable devices (eg, Apple Watch, Fitbit, Garmin, and biosensors). Our participants were sourced through the National Federation of the Blind and a community rehabilitation center located in the Urbana-Champaign area. To qualify for this study, participants had to meet the following criteria: (1) aged 50 years or older; (2) living with visual impairment (ie, low vision or blindness); (3) proficiency in speaking and reading English; (4) uses mHealth apps or wearables for health and well-being; and (5) residing in the United States. Participants with significant cognitive impairments affecting communication were not included in this study. Interested individuals completed a web-based screening questionnaire to determine their eligibility. Those who met the inclusion criteria were then invited for Zoom interviews, scheduled at their preferred times.

### Data Collection

To understand the characteristics of each participant, we collected the following background information at the beginning of interviews: gender, age, type of visual impairment, onset of visual impairment, and perceived health conditions. A comprehensive interview protocol was developed during multiple research team meetings to ensure clarity and focus on the purpose of this study. This interview protocol was then pilot tested with 3 older adults who have low vision to gauge its readability and understandability. Feedback from these pilot interviews was instrumental in refining the wording and phrasing of the questions. The principal investigator of this study, with extensive expertise in qualitative research, used the Zoom conferencing tool to conduct all the interviews, ensuring consistency in the interviewing approach. Prior to each session, participants were informed about the purpose of the interview and their rights. With their verbal consent, we recorded each Zoom interview for accurate data capture and transcription. The interviews were in-depth, with each session averaging about 70 minutes in duration. The data collection phase spanned from June 1 to July 31, 2023, during which we successfully conducted a total of 7 interviews.

### Data Analysis

The recorded interviews were transcribed verbatim into digital format, ensuring the accuracy and integrity of the participants’ responses. These transcriptions were then distributed among the research team members for independent coding. The research team used Google Docs to share interview scripts and conduct an independent coding. To analyze the data systematically, we employed the thematic analysis method [17]. This approach allowed us to structure and identify salient themes that emerged during each researcher’s initial open coding phase. Taking insights from the literature review including the selected theoretical model [12], we integrated the preliminary codes with emerging themes, which led us to a second, more focused round of coding. Following this, the various coding outcomes were aggregated, clustered, and categorized during a comprehensive team analysis session. In instances where there were discrepancies or differences in interpretations between researchers, these were thoroughly addressed and resolved through group discussions, ensuring a unified and cohesive understanding of the qualitative data. The findings from the thematic analysis were communicated to the participants via email; however, no feedback was received.

## Results

### Participant Background Information

Of the participants who completed the Zoom interviews, 3 were men and 4 were women, aged between 53 and 70 (mean 58.43, SD 6.48) years (Table 1). The racial distribution included 2 African Americans and 5 Whites. All participants had pursued higher education, with one holding a bachelor’s degree in computer science. Using the ICD-10 (International Classification of Diseases, 10th version) as a reference for visual impairment criteria, 3 participants identified as having low vision and 4 as blind. Low vision is defined by a visual acuity between 0.05 (20/400) and 0.3 (20/60) or a central visual field of 10 to 20 degrees. Conversely, blindness is indicated by a visual acuity below 0.05 (20/400) or a central visual field under 10 degrees [18,19]. The duration living with visual impairment ranged from 4 to 55 (mean 30.43, SD 19.46) years. In terms of self-assessed computer or smartphone proficiency levels, 2 participants considered themselves experts, 2 as advanced, and 1 as basic. Except for 1 participant rating their familiarity with smartphone apps and wearables at a moderate level (2 on a scale from 0 to 5), all others identified as advanced (4 out of 5).

Table 2 summarizes information on participants’ self-rated overall health, health-related metrics they monitor, and the mHealth technologies they employ or have employed for this purpose. Participants who exercised daily tracked their workouts and aimed to meet their set goals. All of them monitored their daily activities, noting their walking distance, step count, and sitting time. They were keen on both the quantity and quality of their sleep, routinely checking the number of hours they slept each night. In total, 6 used Apple Watches and iPhones, often using Apple Health or Apple Fitness apps. One used a Samsung Galaxy smartphone.

**Table 1:** Characteristics of participants (N=7).

ID	Age (years)	Gender	Race or ethnicity	Education	Visual impairment
1	70	Man	White	Bachelor’s degree	Blindness with light perception (acquired 20 years), both eyes
2	55	Man	White	Bachelor’s degree	Blindness without light perception (congenital), both eyes
3	54	Woman	White	Bachelor’s degree	Blindness without light perception (congenital), both eyes
4	53	Woman	African American	Bachelor’s degree	Low vision (acquired 40 years), both eyes
5	63	Woman	African American	Bachelor’s degree	Low vision (acquired 20 years), both eyes
6	61	Man	White	Bachelor’s degree	Blindness with light perception (acquired 20 years), both eyes
7	53	Woman	White	Bachelor’s degree	Low vision (acquired 4 years), both eyes

**Table 2:** Characteristics of participants (N=7).

ID	Self-rated overall health (1-10; 1 = Not good, 10 = Very good)	Interested in health topics	Selected mHealth^a^ technology tools
1	8	Sleep, general health, exercise, step count, body weight, body temperature, oxygen saturation level, blood pressure	Wearables: Apple Watch mHealth apps: MyChart, SleepWatch, Apple Fitness Others: Talking scale
2	9	Walking distance mile, heart rate, body weight, calorie intake	Wearables: Garmin, Apple Watch mHealth apps: MyFitnessPal Others: Talking scale
3	10	Exercise, menstrual cycle	Wearables: Apple Watch mHealth apps: Apple Fitness
4	7	General health, step count	Wearables: Apple Watch mHealth apps: Apple Fitness, Apple Health Others: Telehealth services
5	5	Physical activity, exercise, heart rate, blood pressure	Wearables: Fitbit mHealth apps: Fitbit
6	8	Sleep, step count, heart rate	Wearables: Apple Watch mHealth apps: Apple Fitness, Apple Health
7	7	Diet, sleep, step count, body weight, blood glucose level	Wearables: Apple Watch, Fitbit mHealth apps: MyChart, Dexcom, MyFitnessPal, Fitbit, Apple Fitness, Apple Health

^a^mHealth: mobile health.

### RQ1. How do Middle-Aged and Older Adults With Low Vision or Blindness Use mHealth Technologies, Including mHealth Apps and Wearable Devices, for Managing Their Health?

#### Purchasing Accessible mHealth Tools

All participants of this study showed a positive attitude toward an mHealth app, or a wearable device usage. In total, 2 participants (ID1 and ID2) proactively sought accessible mHealth apps they are curious about or require. They were technically adept, possessing the knowledge and past job-related experience to use the mHealth app and wearable device autonomously. Others (ID3, ID4, ID5, and ID6) became interested in the mHealth app and wearable device after observing its usage by peers, hearing recommendations or advertisements, or upon a health care professional’s suggestion to address a health problem (ID7). Notably, a unanimous trend emerged: all participants evaluated the quality of the accessibility features of mHealth technologies prior to purchase.

Once it’s installed, then I have to figure out if it is accessible enough, and if it’s not, I just take it off.

ID3

#### Different Types of Visually Impaired mHealth Technology Users

Based upon their attitudes toward using mHealth technologies, we classified them into three types of users: (1) innovative proactives, (2) adaptive users, and (3) trial-and-error adjusters. The individuals who are innovative proactives (ID1 and ID2) tend to enjoy exploring new mHealth technologies, adopt them early, and proactively address and improve any accessibility issues they encounter.

If you teach this stuff, you ask what’s available, and then you do your research. Then, I also have quite a few mentors who are also blind, who have done marathons and things, and so I just kind of follow them and see what works. And then I love to teach and help people.

ID2

The adaptive users (ID5 and ID6) teach themselves to use established technologies and adjust to, and make compromises with, any accessibility issues they come across.

The manual was very easy to understand for me. And I checked their website to know what is up… sometimes, I made a phone call to my friend to ask for help.

ID

The trial-and-error adjusters (ID3, ID4, and ID7) may not actively seek standardized or shared knowledge but learn through trial and error or use workarounds, often compromising on any accessibility issues they encounter.

Just try it. If it does not work, then give up or find another option.

ID

The participants primarily tracked their health data for health promotion and maintenance, rather than medical purposes. Those who exercised daily, such as participants ID1, ID3, and ID5, monitored both the duration and intensity of their activities to achieve their set goals. Sleep consistency was another area of interest, with participants ID1, ID6, and ID7 focusing on maintaining a regular sleep duration by monitoring their nightly rest. Certain participants, including ID2, ID5, and ID6, harnessed mHealth technologies for tailored uses, capturing data related to abnormal heart rates or rhythms on wearables. This was especially pertinent for those with a familial cardiac history or concerns about their cardiac function. Additionally, participant ID2 used a mHealth app to manage her medical records, valuing the confidentiality it offered and reducing the reliance on sighted individuals for tasks such as signing consent forms.

I like to sign all the paperwork before I go into the doctor’s office and not have someone sign it for me. I find that MyChart makes me feel a lot better because it looks at my face, lets me into my health information and tells me what my next appointment is.

ID2

Additionally, participant ID7, diagnosed with diabetes, used a glucometer integrated with a health app to track daily blood sugar levels, following her doctor’s advice.

My doctor recommended this app to monitor my daily glucose levels. Whenever I use the device, it automatically sends the readings to my doctor.

ID7

The regularity and timing of accessing health data via devices or apps were primarily influenced by the tool’s purpose and the user’s preferences. Some participants, namely ID3, ID4, and ID6, habitually checked their apps at specific times each day. In contrast, participants ID1 and ID7 sporadically reviewed health data during the day, while ID5 only did so upon receiving an alarm or notification. Most seldom reviewed historical data, such as trends over extended periods. Only participant ID4 accessed the Apple Health web app monthly to review her personal health data. Typically, the approach was to juxtapose data from the previous day with the current one, or to compare daily figures against an average.

I checked my health data like my exercise minutes, my movement overall, probably 10 times a day at least. I’m constantly looking at it. As a matter of fact, I’m looking at it right now.

ID1

### RQ2. What Are the Obstacles That Middle-Aged and Older Individuals With Low Vision or Blindness Face When Using mHealth Apps and Wearable Devices?

#### Inaccessible User Manuals

When inquiring about their experiences learning to use mHealth technologies, 4 participants highlighted the inaccessibility of user manuals. Despite their desire to access digital manuals, either directly from websites or downloaded versions, their screen readers could not read the digitized information.

I want to have some kind of a step by step instruction on how to navigate using voiceover to be able to accomplish specific tasks like being able to look at my oxygen levels for the past week and in the health app. They do it visually but they don’t do it for people who can’t see.

ID1

In total, 1 participant highlighted that user manuals, as they are currently published, offer limited utility for individuals with visual impairments unfamiliar with assistive technology.

There are two types of blind voiceover users: those that are learning the product and those that already know how to use the product, the main screen reader functionality. So I would say for people that have never used an iPhone before, learning how to use it from a manual is not a good idea.

ID4

Meanwhile, when participants who are advanced in digital technology faced difficulties with the latest products or apps, they contacted the rehabilitation center’s assistive technology specialist. Yet, this individual was not consistently able to provide answers to their questions.

Local vocational rehabilitation workers are not that expert-“They just basically said how to turn on the computer and how to select an application and get it up. But they didn’t really kind of go into more detail than that, and maybe that was the level of the kind of questions they got and that was all the expertise they had.”

ID1

#### Not Visually Impaired-Friendly User Interfaces

The second challenge arose when they navigated mHealth app interfaces. Depending on the design of mHealth apps, screen readers could not directly convert visual symbols or images into audible information, limiting the participants’ ability to interact with content via icons in the same way sighted users can.

Maybe the visual information, sometimes it’s hard for me to even see most of the visual cues. Sometimes they’re not even accessible through screen readers. The screens are also small. So it’s hard when I touch it to get some things to navigate.

ID4

#### Inaccessible Health Data Visualizations

The apps’ health data visualizations were not designed to work with screen readers. For instance, most charts, and graphs were skipped by screen readers without providing any audio descriptions. This makes it challenging for participants to engage with and gather health data from these visualizations. Several participants require more time to engage with charts and graphs, and they often extract information less accurately than sighted users.

Just because I can see numbers on the crack, and hear numbers on the screen doesn’t mean it’s accessible, you know, especially in a table.

ID1

Occasionally, some graphs incorporated sound effects, however, the health information conveyed through the pitch was frequently superficial or meaningless.

They give it to you as an audio graph, and it’s like the intensity of the noise changes based on time. And some people don’t know how to interpret the sound effects over time. It’s not so intuitive.

ID6

#### Unintuitive Arrangement of mHealth App Content

Some participants with blindness frequently struggled with the app’s menu structure and locating desired information. When the app presented information in a convoluted and nonintuitive hierarchy, they had trouble recalling the steps they took to access specific details.

The difficult part for me was finding what sections Apple put different information. So that really wasn’t a voiceover accessibility issue. But it was an accessibility issue, and not knowing where to go to find the information… I don’t always know where to look for information. But again, like not always knowing where to look for things can be challenging. [ID6]

#### Cryptic Health Numbers in Need of Interpretation

Many study participants indicated that they only acknowledge the primary health data they reviewed on a daily basis. While apps like Apple’s and Fitbit’s offer an extensive array of numerical health metrics, participants expressed the need for clearer explanations and interpretations of these figures. Instead of just raw numbers and normal ranges, they desired an app that can articulate their health status in a comprehensible manner and provide actionable recommendations based on it.

Yeah, it would be great. Not just being provided with the bar graphs, but with some explanations.

ID5

#### Cognitive Strain From Excessive Audible Information

Ironically, researchers found that when the screen reader provided too much information, it became a source of stress for participants. While they favored meaningful audible information to make up for the loss of text-based content, excessive or irrelevant details disrupted their focus and added to their cognitive load. This mental and cognitive condition impacts their sustained use of mHealth technology, as the excessive spoken information leads to increased fatigue and stress.

There’s plenty of data that I don’t understand the health aspect of. But I can usually understand everything that’s being reported to me like… It’s a bunch of information bunched together and the voiceover reads it all at the same time, but it’s not clear what everything refers to. So that took a few times to get used to and just read it segment by segment and have someone read me what it says on the screen.

ID6

#### Skepticism Regarding the Accuracy of Health Data

Further, 2 participants (ID5 and ID6), both aged older than 60 years, expressed doubts about the accuracy of the health data presented to them. For instance, participant ID5 mentioned that she believed she took more daily step counts than what the app indicated. These 2 participants believed that their wearable devices’ physical activity measurements were inaccurate. However, they still gauged whether they walked more or less than the previous day based on the fluctuations in their daily readings. Despite their skepticism about the accuracy, they continued using their devices.

Well, I don’t think calorie count is ever accurate, but that has nothing to do with the device. It’s consistent, but it might be consistently off, that’s all.

ID6

### RQ3. How do mHealth Apps and Wearable Devices Impact the Health and Health Management of Middle-Aged and Older Adults With Low Vision or Blindness?

#### Discomfort From Context-Ignored mHealth App Suggestions

All apps installed and used by participants featured prompts or alarms that failed to consider the user’s specific context. For instance, a participant using an app to reduce prolonged sitting and enhance her sedentary lifestyle was frustrated by the app’s alarm. These alarms frequently overlooked her moments of immobility. Furthermore, when a wearable device unexpectedly activated a haptic alarm for some reasons, participants expressed confusion, often uncertain about the alarm’s intent or meaning.

They do not consider the context. I’d say I’m slightly dissatisfied because they’re a little bit judgy. I’m sitting there talking to friends and it nudges me, hey you didn’t move. Well, no, I’m in a conversation, or you need to stand. I’m in the bathroom right now. No, I can’t.

ID3

#### Health Behavior Goal Achievement

Participants conveyed a feeling of accomplishment and satisfaction when they met their health behavior goals. They deliberately participated in physical activity to achieve their daily exercise targets. Moreover, they felt comforted when their health indicators stayed within their expected range, underscoring their dedication to a healthy way of life.

It’s the Apple Watch and you know I track my rings. Have the rings on there, and they have movement exercise. So, and then they set goals and then they give you a goal for the month to hit… Exercise isn’t tracking my exercise because it gives me some kind of goals to achieve so not only looking at my current status, but also helping me to keep goals and keep doing preventative stuff as well.

ID1

It changed my behavior in a lot of different ways. Just having the information is kind of an incentive for me to not want to. I want to accomplish those goals everyday… I’m proud of myself when I get my rings done.

ID3

#### Self-Imposed Pressure on Personal Goals

Frequently, participants expressed feeling overwhelmed and stressed when unable to meet their health goals. For instance, if they did not achieve their target of 10,000 steps a day, it led to feelings of disappointment and frustration. They would habitually check their Apple Watch for progress updates, and the absence of goal achievement notifications became a source of unease, compelling them to keep glancing at it. Further, 1 participant mentioned that flexibility enables them to lead a healthy lifestyle daily without feeling stressed.

Pressure… Anxiety… There is, you know, pressure to do that and maybe you kind of get a little anxious. Oh, man, if I am not gonna hit my targets. There needs to be a little bit of flexibility that way. I guess that there’s some pressure that people put on themselves.

ID1

#### Immediate Response to Health Red Flags

In total, 2 participants shared their experiences, noting that mHealth apps played a pivotal role in alerting them to urgent health situations. Furthermore, 1 participant (ID5), with a family history of heart disease, expressed relief in being able to promptly notify his doctor upon detecting an irregular or abnormal heart rhythm. Another participant (ID7) became alarmed when her glucose level spiked, necessitating a prompt response.

I’m checking my heart rate and if I see an abnormal number or see I’m not so healthy, what I do is I just raise an issue. I call a physician and then ask what’s the problem? And then he calls me over if it’s something serious.

ID5

#### Self-Reflection and Behavioral Adjustments

The insights from their mHealth apps and wearables attuned them to their bodies and the refreshed health data guided their subsequent health behaviors. The feedback from the mHealth app offered a baseline for them to adjust their health behaviors. They expressed that reflecting on these behaviors spurred them to adopt healthier practices.

I like my Apple Watch because it tells me how many calories I burned. In fact, it cut me down a little bit because I knew I wasn’t moving as much as I thought I was. And I’m okay with that. But at the same time, I want to get more moving and walking and hanging out with friends and just being part of life. I enjoy it very much.

ID3

It doesn’t motivate me but keeps me on track and tells me if I’ve not done so enough.

ID5

I can see how many hours I slept and make adjustments based on that. What time I go to sleep, the time I wake up, and then for swimming, I would look at things like heart rate and decide how much harder the next day I should swim or not.

ID6

## Discussion

### Principal Findings

Contrary to our initial presumption that their visual impairments and aging would lead to more diverse difficulties in using mHealth technology, our findings mirrored previously reported accessibility and usability issues observed in younger visually impaired individuals. The usage of mHealth apps and wearable devices among participants, contingent on the severity of their visual impairment, began with an assessment of the accessibility features of these tools prior to purchase. They exhibited 3 distinct usage patterns: innovative proactives, adaptive users, and trial-and-error adjusters. The research team identified several barriers, including (1) a scarcity of accessible user manuals, (2) user interfaces that are not visually impaired-friendly, (3) health data visualizations that are not accessible, (4) unintuitive arrangement of app content, (5) health information that is challenging to comprehend, (6) cognitive overload caused by an excess of audible information, and (7) skepticism regarding the accuracy of health records. Despite these challenges, the use of mHealth apps and wearable devices has influenced health management, although sometimes participants experienced discomfort due to feedback that lacked context sensitivity. Nevertheless, the ability to track and monitor health metrics served as a motivation for health management and as a form of recognition for their efforts.

Similar to Davis’ [20] technology acceptance model, the factors influencing the use of mHealth technology among participants were largely determined by their perceptions of the technology’s usefulness and benefits. The accessibility related features of mHealth apps and wearable devices significantly influenced the participants’ decisions to purchase products rather than health management related features. Furthermore, they often tolerate the inconveniences associated with mHealth apps and wearable devices due to their interest in the purposes they serve. This behavior can be attributed to their greater adaptability and accommodating nature. Future research is required to ascertain whether temperamental changes due to psychological maturation in older individuals drive their persistence in using inaccessible technologies, or if the technological aids assisting their daily lives lead them to overlook accessibility challenges. Understanding the determinants of technology usage in older populations can not only shape strategies to enhance technology demand but also offer insights into the technology development phase [21].

Based on the learning patterns observed in the adoption of mHealth technologies, we categorize users into three types: (1) innovative proactives, (2) adaptive users, and (3) trial-and-error adjusters. Individuals in the “Innovative Proactives” category are enthusiastic about the novelty of emerging technologies and proactively seek ways to adapt to them. They frequently consult information technology company websites, specialized web-based communities like AppleVis [22], and YouTube tutorials to learn how to navigate interfaces. Over half of the participants expressed that the user manuals supplied by the manufacturers were not tailored to their needs—a finding not highlighted in prior studies. These PDF manuals were frequently inaccessible, making it challenging to grasp the content without visualizing the app’s layout. Future research is needed to understand whether user-friendly manuals can influence the learning process of users who typically resort to workarounds or only use technology in ways they are familiar with. Further, individuals with visual impairments who possess advanced skills in screen readers often find limited resources from rehabilitation centers about digital tools. They are experts in screen readers and can sometimes identify critical accessibility issues in systems, guiding software developers toward solutions. Since these individuals with visual impairments have deep knowledge and expertise in digital technology accessibility [23], it is important to use them as educational resources for other visually impaired individuals. By doing so, we not only value their contributions but also foster their connection to visual impairment communities, enabling them to aid others in the technology adoption field.

Health data visualizations in mHealth apps offer numerous benefits to individuals who are interested in their health data. They simplify complex health data interpretation for individuals, aiding in spotting patterns and trends that can improve understanding health conditions and support health behavior changes [24]. Specifically, such visualizations promote self-care in chronic disease management [25]. Health data visualizations are pivotal in extracting insights from mHealth apps and wearables, facilitating personalized health strategies and research advancement [26,27]. However, upon reviewing past research on health data visualization for older adults within the realm of mHealth or web apps, the research team found no studies directly addressing this topic. Research is still limited on how visually impaired older adults access, interpret, and are influenced by visualized health data in their health management. A participant with mild visual impairment, who relies on magnifiers to see objects, found health data visualizations beneficial for understanding weekly sleep trends, citing the Fitbit sleep chart as particularly effective. However, participants with blindness, who use screen readers, indicated that the audio descriptions from visualizations were insufficient, offering only discrete numbers without added context. Thus, the severity of visual impairment seems to influence the effectiveness and impact of health data visualizations on users. To amplify the impact of health data visualization in mHealth apps and improve the health literacy of older adults with visual impairments, more research into inclusive health data visualization is essential.

All participants used Apple Watches and Fitbits for their health management. These wearables inform users about health issues via on-device alerts and companion apps. They display reminders for medication, movement prompts, and abnormal heart rate readings. In addition, they provide real time updates on health metrics such as step counts, sleep patterns, and alert users about events like achieving an activity goal or informing a detected abnormal heart rhythm. Previously, Cadmus-Bertram et al [28] verified the positive effect of the Fitbit alarms on physical activity levels. However, it remains unclear which mode can effectively inform visually impaired individuals. Participants often did not recognize the reason for their wearables’ haptic alert. They typically had to access a smartphone app to discern the nature of these sporadic alerts, and even then, sometimes remained unsure of the alert’s meaning. Visually impaired users should be given a prior explanation of an alert’s purpose and mechanism on their mobile device to ensure they can comprehend and act upon it. In a similar vein, recent mHealth-based intervention studies highlight the use of health behavior change techniques, emphasizing personalized feedback and the advancement toward more interactive and timely delivery of personal health information [29]. As highlighted by Krishna et al [30], message frequencies in mHealth systems can differ significantly, from as often as 5 times daily for diabetes management and smoking cessations, to merely once a week for encouraging physical activity [31]. The uncertain impact of message frequencies in mHealth could jeopardize the continuous and timely support required by individuals with health care needs.

In the context of adopting mHealth technologies, the impact of age-related cognitive decline on older adults is an important consideration. As people age, they often experience declines in several cognitive functions, including processing speed, working memory, inhibitory function, long-term memory, attention, and problem-solving abilities [32]. In addition to their limited experience with technology and unfamiliarity with smartphone apps, cognitive decline in memory and attention challenges them in remembering and multitasking in complex apps [33]. One of the identified obstacles was that they struggled to recall the path they took to access specific personal health information or app features. In particular, when an app’s menu contains multiple submenus or necessitates clicking on small icons or hyperlinked text, participants reported spending significant time navigating the app. Czaja [7] noted that navigation challenges for older users arise from cognitive and perceptual demands. They emphasized the importance of tailoring app development to accommodate these users’ needs and preferences. Creating more intuitive and user-friendly interfaces for visually impaired older adults can enhance the use of mHealth technologies, thereby improving the efficacy and effectiveness of self-care.

As mHealth apps continue to expand their features, it is imperative to ensure these tools cater to the unique needs of older populations. Specifically, considering factors such as visual impairment, cognitive decline, and poor technological literacy can make these apps more accessible and effective for older individuals [34]. When inquiring about the preferred number of mHealth app features or menus, 3 participants favored fewer features, while 4 opted for a diverse range of features to cater to their varied interests and needs. The participants of this study, while appreciating the app’s diverse features or menus, expressed discomfort with its complexity. They suggested that a simplistic design with limited features might not necessarily enhance the satisfaction of older users with visual impairments. This implies that allowing customization of features, menus, and their organization within apps could enhance the user experience with personal informatics systems.

Prior research on blind individuals’ voice interactions with smartphones has predominantly centered on the usage of voice assistants and commands to improve accessibility and usability for those with visual impairments [35]. Voice assistants and commands offer audio feedback, aid in navigation, and execute diverse tasks on smartphones, thus improving accessibility and usability for users with visual impairments. While voice assistants offer potential benefits for visually impaired older adults due to their straightforward speech-based interactions, there is limited understanding of how this demographic perceives and reacts to communicating with a device lacking a graphical user interface. Given the limited prior research on the topic, 1 study delving into the potential of smart speaker voice assistants (eg, Amazon Alexa, Google Assistant, Apple’s Siri, Microsoft’s Cortana, and Samsung’s Bixby) to assist older adults with their health and independence emphasized the simplicity and speech-focused interaction of these voice assistants [36].

Training opportunities in mHealth technologies are essential for older populations to ensure their effective and purposeful usage. Although research on training programs for older adults is limited, some studies have examined the effects of technology training on their experiences with mHealth technologies [37,38]. To offer training opportunities in mHealth for older individuals, Rodgers et al [39] emphasized the importance of instructing health professional students to interact with them. They posited that intergenerational training programs hold promise in enhancing older adults’ skills with mHealth technologies. Furthermore, there is a need for learning opportunities tailored to visually impaired individuals who regularly use the accessibility features of digital technologies. Given the variety of disabilities and chronic conditions present in the older population, future research should focus on effective methods for instructing them in the use of new mHealth technologies for their health management.

So far, numerous efforts have been made to improve web information access for older populations, notably through guidelines tailored for older users’ website design [40]. Yet, there is a noticeable absence of mHealth app design guidelines for researchers and software developers. Furthermore, we found no guidelines specifically developed for mHealth technology usage by individuals with low vision or blindness. This lack of attention may exacerbate health disparities and future health care inequalities. While the MOLD-US framework [12] sheds light on how aging or chronic condition barriers impact mHealth technologies, the relationships and interactions between the components affecting this population’s mHealth usability remain unclear. Even as the framework aids in assessing older individuals’ mHealth technology adoption, there is a need for deeper insight into which factors developers and researchers should prioritize for optimal digital health care support. At this juncture, the results of this study, which centers on visual impairment in middle-aged and older individuals, are anticipated to enhance our understanding of their mHealth technology usage. These insights hold significance for guiding future research within this distinct population.

### Limitations

The insights from this qualitative study, based on interviews with a limited group of participants, may not reflect the experiences of all middle-aged and older visually impaired individuals. Given the limited previous research on older individuals’ use of mHealth technology, this exploratory study aimed to highlight the need to design technologies that don’t inadvertently exacerbate health disparities in our digitally divided aging society. To better understand the varied mHealth technology experiences across a larger demographic, future research should consider a more extensive sample and the benefits of quantitative methods. Moreover, since participants in this study generally reported good health, there is a gap in knowledge about how older individuals with visual impairments use mHealth technology in contexts requiring medical consultations and clinical care. In summary, it is worth noting that this study was a cross-sectional interview study with a limited number of cases.

### Conclusions

In conclusion, middle-aged and older adults with visual impairments incorporate mHealth apps and wearables into their daily health management. Their familiarity with and attitudes toward technology seem to influence their usage of mHealth solutions. By focusing on their personal health data of interest, they establish daily health routines, setting self-driven goals and modifying health behaviors accordingly. While challenges such as inaccessible user interfaces, overwhelming features, and complex content structures exist, they frequently use their preferred functionalities and tend to disregard the inaccessible ones. The ease of use and satisfaction derived from these technologies greatly influence their usage among older users. It is imperative that the provided health data, visualizations, and feedback are designed with the individual’s health literacy and context in mind. The accuracy and sensitivity of voice interaction systems appear to enhance mHealth usage for this demographic. Finally, there is a clear need for more accessible user manuals and increased training opportunities.

## Abbreviations

ICD-10: International Classification of Diseases-10th version
mHealth: mobile health
MOLD-US: mHealth for Older Users

## Data Availability

The interview data generated or analyzed during this study are available from the corresponding author on reasonable request.

## References

[1] Corn AL, Erin JN (2010). Foundations of Low Vision: Clinical and Functional Perspectives.

[2] Bourne RRA, Stevens GA, White RA, Smith JL, Flaxman SR, Price H, Jonas JB, Keeffe J, Leasher J, Naidoo K, Pesudovs K, Resnikoff S, Taylor HR (2013). Causes of vision loss worldwide, 1990-2010: a systematic analysis. Lancet Glob Health.

[3] Park D, Gutchess A (2006). The cognitive neuroscience of aging and culture. Curr Dir Psychol Sci.

[4] Chan T, Friedman DS, Bradley C, Massof R (2018). Estimates of incidence and prevalence of visual impairment, low vision, and blindness in the United States. JAMA Ophthalmol.

[5] (2011). mHealth: new horizons for health through mobile technologies: second global survey on eHealth. WHO Global Observatory for eHealth.

[6] El-Rashidy N, El-Sappagh S, Islam SMR, El-Bakry HM, Abdelrazek S (2021). Mobile health in remote patient monitoring for chronic diseases: principles, trends, and challenges. Diagnostics (Basel).

[7] Czaja SJ (2016). Long-term care services and support systems for older adults: the role of technology. Am Psychol.

[8] Caldeira C, Chen Y, Sayago S (2019). Seniors and self-tracking technology. Perspectives on Human-Computer Interaction Research with Older People. Human–Computer Interaction Series.

[9] Bhattacherjee A (2001). Understanding information systems continuance: an expectation-confirmation model. MIS Q.

[10] Aranha M, James K, Deasy C, Heavin C (2021). Exploring the barriers and facilitators which influence mHealth adoption among older adults: a literature review. Gerontechnology.

[11] Hong S, Thong JY, Tam KY (2006). Understanding continued information technology usage behavior: a comparison of three models in the context of mobile internet. Decis Support Syst.

[12] Wildenbos GA, Peute L, Jaspers M (2018). Aging barriers influencing mobile health usability for older adults: a literature based framework (MOLD-US). Int J Med Inform.

[13] Petrovčič A, Boot WR, Burnik T, Dolnicar VV (2019). Improving the measurement of older adults' mobile device proficiency: results and implications from a study of older adult smartphone users. IEEE Access.

[14] Wang J, Du Y, Coleman D, Peck M, Myneni S, Kang H, Gong Y (2019). Mobile and connected health technology needs for older adults aging in place: cross-sectional survey study. JMIR Aging.

[15] Chandrasekaran R, Katthula V, Moustakas E (2020). Patterns of use and key predictors for the use of wearable health care devices by US adults: insights from a national survey. J Med Internet Res.

[16] Olmedo-Aguirre JO, Reyes-Campos J, Alor-Hernández G, Machorro-Cano I, Rodríguez-Mazahua L, Sánchez-Cervantes JL (2022). Remote healthcare for elderly people using wearables: a review. Biosensors (Basel).

[17] Sundler AJ, Lindberg E, Nilsson C, Palmér L (2019). Qualitative thematic analysis based on descriptive phenomenology. Nurs Open.

[18] Choi YJ, Kim M, Park KH, Kim DM, Kim SH (2014). The risk of newly developed visual impairment in treated normal-tension glaucoma: 10-year follow-up. Acta Ophthalmol.

[19] Dandona L, Dandona R (2006). Revision of visual impairment definitions in the International Statistical Classification of Diseases. BMC Med.

[20] Davis FD (1989). Perceived usefulness, perceived ease of use, and user acceptance of information technology. MIS Q.

[21] Queiroz MM, Wamba SF, Jabbour CJC, de Sousa Jabbour ABL, Machado MC (2022). Adoption of industry 4.0 technologies by organizations: a maturity levels perspective. Ann Oper Res.

[22] (2023). AppleVis.

[23] Ferreira SBL, da Silveira DS, Capra EP, Ferreira AO (2012). Protocols for evaluation of site accessibility with the participation of blind users. Procedia Comput Sci.

[24] Klasnja P, Pratt W (2012). Healthcare in the pocket: mapping the space of mobile-phone health interventions. J Biomed Inform.

[25] Hassen DB (2020). Mobile-aided diagnosis systems are the future of health care. East Mediterr Health J.

[26] Polack PJ, Chen ST, Kahng M, DE Barbaro K, Basole R, Sharmin M, Chau DH (2018). Chronodes: interactive multifocus exploration of event sequences. ACM Trans Interact Intell Syst.

[27] Hullman J, Diakopoulos N (2011). Visualization rhetoric: framing effects in narrative visualization. IEEE Trans Vis Comput Graph.

[28] Cadmus-Bertram LA, Marcus BH, Patterson RE, Parker BA, Morey BL (2015). Randomized trial of a Fitbit-based physical activity intervention for women. Am J Prev Med.

[29] Hekler EB, Klasnja P, Riley WT, Buman MP, Huberty J, Rivera DE, Martin CA (2016). Agile science: creating useful products for behavior change in the real world. Transl Behav Med.

[30] Krishna S, Boren SA, Balas EA (2009). Healthcare via cell phones: a systematic review. Telemed e-Health.

[31] Zhao SZ, Weng X, Luk TT, Wu Y, Cheung DYT, Li WHC, Tong H, Lai V, Lam TH, Wang MP (2022). Adaptive interventions to optimise the mobile phone-based smoking cessation support: study protocol for a Sequential, Multiple Assignment, Randomised Trial (SMART). Trials.

[32] Park DC, Reuter-Lorenz P (2009). The adaptive brain: aging and neurocognitive scaffolding. Annu Rev Psychol.

[33] Peek STM, Luijkx KG, Rijnaard MD, Nieboer ME, van der Voort CS, Aarts S, van Hoof J, Vrijhoef HJM, Wouters EJM (2016). Older adults' reasons for using technology while aging in place. Gerontology.

[34] Thornton LK, Kay-Lambkin FJ (2018). Specific features of current and emerging mobile health apps: user views among people with and without mental health problems. mHealth.

[35] Satra T, Shah M, Lad A, Correia S, Jacob IJ, Falkowski-Gilski P, Shanmugam SK, Piramuthu S (2021). Voice and gesture based app for blind people. Data Intelligence and Cognitive Informatics: Proceedings of ICDICI 2020.

[36] Kim S (2021). Exploring how older adults use a smart speaker-based voice assistant in their first interactions: qualitative study. JMIR mHealth uHealth.

[37] Lai WX, Visaria A, Østbye T, Malhotra R (2023). Prevalence and correlates of use of digital technology for managing hypertension among older adults. J Hum Hypertens.

[38] Pihlainen K, Korjonen-Kuusipuro K, Kärnä E (2021). Perceived benefits from non-formal digital training sessions in later life: views of older adult learners, peer tutors, and teachers. Int J Lifelong Educ.

[39] Rodgers J, Segal-Gidan F, Reilly JM (2022). Impact of an interprofessional health student education program on older adult participants. Gerontol Geriatr Med.

[40] Hanson VL (2001). Web access for elderly citizens.

